# Prefrontal oxygenation correlates to the responses in facial skin blood flows during exposure to pleasantly charged movie

**DOI:** 10.14814/phy2.13488

**Published:** 2017-11-09

**Authors:** Kanji Matsukawa, Kana Endo, Ryota Asahara, Miho Yoshikawa, Shinya Kusunoki, Tomoko Ishida

**Affiliations:** ^1^ Department of Integrative Physiology Graduate School of Biomedical and Health Sciences Hiroshima University Hiroshima Japan

**Keywords:** Cerebral prefrontal cortex, emotional challenges, facial skin blood flow, hand skin blood flow, near‐infrared spectroscopy

## Abstract

Our laboratory reported that facial skin blood flow may serve as a sensitive tool to assess an emotional status. Cerebral neural correlates during emotional interventions should be sought in relation to the changes in facial skin blood flow. To test the hypothesis that prefrontal activity has positive relation to the changes in facial skin blood flow during emotionally charged stimulation, we examined the dynamic changes in prefrontal oxygenation (with near‐infrared spectroscopy) and facial skin blood flows (with two‐dimensional laser speckle and Doppler flowmetry) during emotionally charged audiovisual challenges for 2 min (by viewing comedy, landscape, and horror movie) in 14 subjects. Hand skin blood flow and systemic hemodynamics were simultaneously measured. The extents of pleasantness and consciousness for each emotional stimulus were estimated by subjective rating from −5 (the most unpleasant; the most unconscious) to +5 (the most pleasant; the most conscious). Positively charged emotional stimulation (comedy) simultaneously decreased (*P *<* *0.05) prefrontal oxygenation and facial skin blood flow, whereas negatively charged (horror) or neutral (landscape) emotional stimulation did not alter or slightly decreased them. Any of hand skin blood flow and systemic cardiovascular variables did not change significantly during positively charged emotional stimulation. The changes in prefrontal oxygenation had a highly positive correlation with the changes in facial skin blood flow without altering perfusion pressure, and they were inversely correlated with the subjective rating of pleasantness. The reduction in prefrontal oxygenation during positively charged emotional stimulation suggests a decrease in prefrontal neural activity, which may in turn elicit neurally mediated vasoconstriction of facial skin blood vessels.

## Introduction

One empirically knows that anger or embarrassment evokes not only specific facial expression but also flushing. Facial skin blood vessels are richly innervated by both sympathetic and parasympathetic nerves (Kuchiiwa et al. 1992; Drummond 1994). Facial parasympathetic nerves may have a vasodilator function, because stimulation of the chorda tympani and lingual nerves increased feline lip skin blood flow (Izumi and Karita 1993). Lesion of the facial sympathetic pathway diminished the vasodilator response to body heating and vasoconstrictor response to body cooling, suggesting that facial sympathetic nerves may have not only vasoconstrictor but also vasodilator function (Blair et al. 1961; Drummond and Lance 1987). Emotional expression of anger or embarrassment accompanied increased forehead skin blood flow, which was diminished by lesion of the sympathetic pathway (Drummond and Lance 1987). Accordingly, facial skin blood flow may be increased by activation of parasympathetic and sympathetic vasodilator nerves and partly by withdrawal of sympathetic vasoconstrictor activity. We have recently examined the effects of emotionally charged stimulation on regional facial skin blood flow and conductance with noninvasive two‐dimensional laser speckle flowmetry (Matsukawa et al. 2017). During viewing a positively charged comedy movie, facial skin blood flow and vascular conductance decreased in relation to the subjective rating of pleasantness. In contrast, limb skin blood flow and vascular conductance and systemic hemodynamics showed insignificant or slight changes and none of them correlated with the subjective rating of pleasantness (Matsukawa et al. 2017). Thus, regional facial skin blood flow may serve as a more sensitive tool to assess an emotional status than limb skin blood flow and systemic hemodynamics.

The prefrontal cortex plays an important role in multiple physiological functions such as attention, planning of motor act, cognitive function, and autonomic control. The prefrontal cortex is also engaged in emotional recognition and processing, because emotion recognition was impaired by lesion of the ventromedial prefrontal cortex (Wolf et al. 2016) or stroke of the right middle cerebral artery (Paradiso et al. 2011). It is known that the medial prefrontal cortex has efferent projections to central autonomic structures such as the nucleus tractus solitarius and to sympathetic preganglionic neurons in the thoracic and lumbar spinal cord (Takagishi and Chiba 1991; Chiba et al. 2001; Gabbott et al. 2005; Levinthal and Strick 2012; Dum et al. 2016) and that the electrical and chemical activation of the cortical area evokes cardiovascular, respiratory, and metabolic changes (Verberne 1996; Owens and Verberne 2000, 2001; Hassan et al. 2013). Resstel et al. (2006) reported that the medial prefrontal cortex is involved in behavioral and cardiovascular responses to fear conditioning in conscious rats. Neuroimaging human studies with positron emission tomography (PET) and functional magnetic resonance imaging (fMRI) suggest that prefrontal activity correlates with heart rate variability and the cardiovascular responses during arousal or emotional stimulation (Critchley et al. 2000; Lane et al. 2009) and cardiac vagal control of heart rate (HR) during exercise (Wong et al. 2007a,b; Norton et al. 2013). Thus, emotional stimulation may elicit modulation in the prefrontal activity, which in turn causes the autonomic and cardiovascular responses.

For real‐time monitoring of cortical activity in a less restrained and less stressful condition, near‐infrared spectroscopy (NIRS) has been developed so as to detect the dynamic changes in regional cerebral oxygenation and deoxygenation. Unfortunately, the previous NIRS data of prefrontal activity during emotional interventions were quite controversial (Bendall et al. 2016). Prefrontal oxygenated hemoglobin (Oxy‐Hb) increased during viewing negatively charged pictures or fearful facial expressions (Marumo et al. 2009; Glotzbach et al. 2011; Hosseini et al. 2011; Ozawa et al. 2014). Curiously, prefrontal Oxy‐Hb also increased during viewing positively charged pleasant pictures (Kreplin and Fairclough 2013) or did not change significantly (Hosseini et al. 2011). Herrmann et al. (2003) found no significant prefrontal effects of emotional interventions by viewing negative and positive pictures or facial expressions. Hoshi et al. (2011) reported considerable intersubject variation in the prefrontal NIRS responses, irrespective of viewing negative or positive pictures. The variant observations may result from how to induce emotion (i.e., viewing emotionally charged pictures or facial expressions) and from a substantial difference in the subjective response to a given emotional intervention. Especially, even if positively charged images are presented, not every subject will experience a pleasant feeling. On the other hand, audiovisually elicited emotional stimulation may more consistently elicit pleasant feelings than viewing positively charged images or facial expressions (Matsukawa et al. 2017). Furthermore, considering that facial skin blood flow may serve as a more sensitive tool to assess an emotional status, prefrontal neural activity should be correlated with the changes in facial skin blood flow during emotional interventions. So we hypothesized that prefrontal neural activity has positive relation to the changes in facial skin blood flow during emotionally charged stimulation. To test the hypothesis, we examined the simultaneous changes in bilateral prefrontal oxygenation and facial skin blood flows during emotionally charged interventions, which were measured with the NIRS and laser speckle and Doppler flowmetry. The correlations of the prefrontal activity to the changes in facial skin blood flows and to the subjective ratings of pleasantness and consciousness were evaluated for the first time.

## Methods

### Subjects

Fourteen healthy volunteers (seven males and seven females) participated in the present study and their anthropologic data are summarized in Table 1. None of the subjects suffered from any known cardiovascular and neuromuscular diseases and they did not take any medication. All procedures and protocols performed in this study were in accordance with the 1964 Helsinki Declaration and its later amendments or comparable ethical standards and were approved by the Institutional Ethical Committee of Hiroshima University (Permit No. 1425). Informed consent was obtained from all individual participants included in the study prior to the experiments. All experiments were performed in thermoneutral and soundproof environment (room temperature, 26 ± 0.2°C; relative humidity, 66 ± 3%).

**Table 1:** Anthropological data and the baseline values of the systemic cardiovascular variables and skin blood flows

(1) Anthropological data (*n* = 14 subjects)	
Age (years)	23 ± 1
Body weight (kg)	58 ± 3
Height (cm)	166 ± 2
Body mass index (kg/m^2^)	21 ± 1
(2) Baseline systemic cardiovascular variables (*n* = 13 subjects)	
HR (beats/min)	68 ± 3
SV (mL)	85 ± 4
CO (L/min)	5.8 ± 0.4
MAP (mmHg)	84 ± 3
TPR (mmHg·min/L)	17 ± 1
(3) Baseline regional facial skin blood flows with laser speckle flowmetry (*n* = 8 subjects)	
Left infraorbital (a.u.)	76 ± 6
Right infraorbital (a.u.)	76 ± 6
Left cheek (a.u.)	74 ± 6
Right cheek (a.u.)	71 ± 4
Lips (a.u.)	144 ± 23
Subnasal (a.u.)	73 ± 7
Chin (a.u.)	68 ± 4
(4) Baseline skin blood flows with laser Doppler flowmetry (*n* = 12–13 subjects)	
Forehead (mL/min/100 g tissue)	10.3 ± 0.85
Left cheek (mL/min/100 g tissue)	12.9 ± 1.99
Left hand (mL/min/100 g tissue)	3.3 ± 0.56

HR, heart rate; SV, stroke volume; CO, cardiac output; MAP, mean arterial blood pressure; TPR, total peripheral resistance; a.u., arbitrary unit.

### Measurements of facial and limb skin blood flows

**Figure 1:**
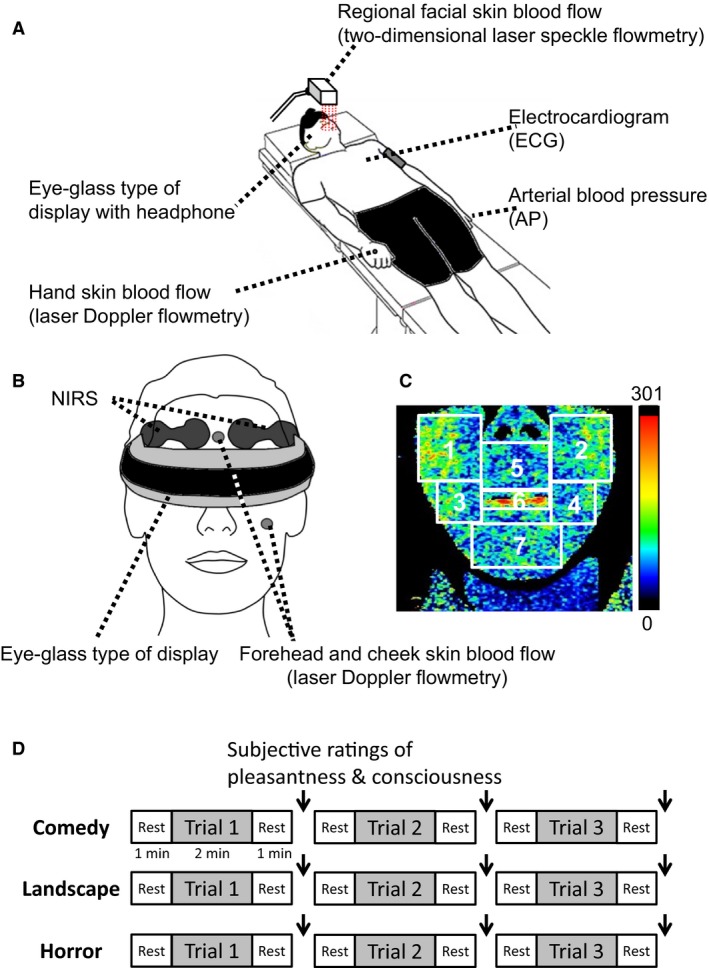
**(**A) An experimental setup. (B) Measurements of bilateral prefrontal oxygenation with near‐infrared spectroscopy (NIRS) and forehead, left cheek, and right hand skin blood flow with laser Doppler flowmetry. (C) An example of two‐dimensional laser speckle measurements of regional facial skin blood flow in seven different facial regions [right (1) and left (2) infraorbital, right (3) and left (4) cheek, subnasal (5), lips (6), and chin (7)]. (D) Protocols about emotionally charged audiovisual stimulation. The subjective ratings of pleasantness and consciousness were asked immediately after each bout of the emotional stimulations as shown by arrows (↓).

Regional facial skin blood flows were monitored in 8 of the 14 subjects over the lower part of the face using a two‐dimensional laser speckle flowmeter with a line‐sensing image device (LFG‐1, Softcare Co., Fukuoka, Japan) as shown in Figure 1. An area of 200 × 200 mm including the face was scanned to the 200 × 200 pixels; the spatial resolution was therefore 1 mm. The time difference between the first and last line images was approximately 12 sec. Forehead, cheek, and hand skin blood flows were monitored with a laser Doppler flowmetry instrument (ALF21, ADVANCE Co., Tokyo, Japan) in 12–13 subjects, whose probes were placed on the forehead, left cheek, and the dorsum of the right hand (Fig. 1). The analog voltage signals of the Doppler skin blood flows were time averaged with a time constant of 0.1 sec.

### Cardiovascular recordings

The cardiovascular responses during exposures to emotionally charged movies were examined in 13 of the 14 subjects. A pair of electrodes (Magnerode, TE‐18M‐3, Fukuda Denshi, Tokyo, Japan) and a ground electrode were attached on the chest for measuring electrocardiogram (ECG). The ECG signal and respiratory movement were monitored with a telemetry system (DynaScope DS‐3140, Fukuda Denshi, Tokyo, Japan). AP was noninvasively and continuously measured with a Finometer (Finapres Medical Systems BV, Arnhem, the Netherlands), of which a cuff was attached to the left middle or index finger. The AP waveform was sampled at a frequency of 200 Hz. The beat‐to‐beat values of mean AP (MAP), HR, cardiac output (CO), stroke volume (SV), and total peripheral resistance (TPR) were calculated from the aortic pressure waveform by using a Modelflow software (BeatScope 1.1, Finapres Medical Systems BV, Arnhem, the Netherlands).

### Oxygenated and deoxygenated hemoglobin concentrations of the prefrontal cortex

The relative concentrations of the oxygenated and deoxygenated hemoglobin (Oxy‐ and Deoxy‐Hb) of the bilateral prefrontal cortices were measured with NIRS in all 14 subjects. A pair of photoemission and photodetection probes were placed on the forehead surface between Fp1 and F3 (left side) and between Fp2 and F4 (right side) (referring to the international EEG 10–20 system) (Elwell et al. 1994; Matsukawa et al. 2015; Asahara et al. 2016) and covered with a black cloth. The interprobe distance was 4 cm. The principle of NIRS is that near‐infrared light from three laser photodiodes with different wavelengths (775, 810, and 850 nm) penetrates brain tissue and some of the light is absorbed by Hb, and that the remaining light scattered by the brain tissue is picked up with photodetectors. The reflected near‐infrared light through brain tissue was sampled at a rate of 6 Hz and converted to optical densities with a near‐infrared spectrometer (NIRO 200, Hamamatsu Photonics, Hamamatsu, Japan). Since the oxygenation signal of NIRS is dependent on a balance between oxygen supply and utilization in microcirculation within the illuminated tissue, the NIRS signal does not directly monitor regional blood flow. Nevertheless, since the Deoxy‐Hb of the prefrontal areas remains at or near the baseline level throughout the interventions, the change in Oxy‐Hb reflects the change in regional tissue blood flow, which may partly follow neural activity in the brain (Fox and Raichle 1986; Hoshi and Tamura 1993; Elwell et al. 1994; Hoshi et al. 2001; Rostrup et al. 2002). Another point to be considered is that the signals of the NIRS are influenced not only by regional cerebral blood flow (rCBF) but also by skin blood flow within the illuminated tissue area. To clarify the possible contribution of skin blood flow to the Oxy‐ and Deoxy‐Hb signals of the NIRS, a laser Doppler flow probe was placed in the middle of the forehead.

### Experimental protocols

Audiovisual stimulation by emotionally charged movies was given to all 14 subjects using the eye glass type of a head‐mount goggle display (HMZ‐T3, Sony Co., Tokyo, Japan) with a headphone, which was controlled by a computer (Fig. 1). The three kinds of movies were selected as emotionally charged stimulation: a comical movie (called “Manzai”) performed by two Japanese comedians, a Japanese horror movie (titled “The Juon” Japanese version Toho, Japan 2000), and a night landscape movie. It was expected that the comical movie might charge the most pleasant feeling, while the horror movie might charge the most unpleasant one and the landscape movie might be neutral. Each of the emotionally charged interventions (comedy, landscape, and horror) consisted of three consecutive bouts involving different scenes captured from an individual movie. The cardiovascular and skin blood flow data were sampled at each bout, which contained the periods before (for 1 min), during (for 2 min), and after movie stimulation (for 1 min). At the pre‐ and poststimulation periods, the subjects watched a small white circle on the black background in the goggles. The data over the 36‐sec period before the onset of emotional stimulation were regarded as the baseline control. A time interval between bouts in a given emotional intervention was approximately 4 min. Immediately after the cessation of each bout, the subjective ratings of pleasantness and consciousness were asked. A transit time interval between emotional interventions (e.g., from comedy to landscape) was approximately 5 min. The order of the three movie interventions was randomized.

Since both prefrontal NIRS signal and forehead skin blood flow decreased in parallel during viewing comedy stimulation in this study, it was important to identify whether or not the prefrontal NIRS signal might simply reflect a fall in facial skin blood flow within the illuminated tissue area. To do this, the changes in forehead skin blood flow were compared with the simultaneous changes in the prefrontal Oxy‐Hb and Deoxy‐Hb during conversation using the identical subjects, because we know that conversation greatly increases prefrontal oxygenation but does not always elicit emotional changes. Conversation was made whenever the subjective ratings of pleasantness and consciousness were asked.

The subjects were asked not to evoke any facial movement during emotional challenges as much as possible, because any skin movement due to facial expressions (such as smiling, laughing, etc.) caused an artifact in laser speckle blood flow measurements. Whenever such facial movement occurred during a trial of the emotional challenges, we could easily notice a big artifact on the laser speckle flow image and remove the data acquired. Thus, the present data collection involved no artifact due to facial expressions during the emotional challenges.

### Subjective ratings of feelings stimulated during emotionally charged movies

The subjective feelings of pleasantness and consciousness were asked immediately after each bout of the emotionally charged stimulation, according to previous studies (Mehrabian and Russell 1974; Mehrabian 1996; Matsukawa et al. 2017). Pleasantness was rated with 11 grades from “the most pleasant” (+5) to “the most unpleasant” (−5). Consciousness was also rated with 11 grades from “the most conscious” (+5) to “the most unconscious” (−5). We explained to the subjects that “the most conscious” means the most awake condition being fully aware of the situation, while “the most unconscious” means the drowsiest or sleepiest condition being fully unaware of the situation. The assessment tables were displayed in the goggles and the ratings were determined according to the feelings by individual subjects.

### Data and statistical analyses

The signals of the prefrontal NIRS and Doppler skin blood flows were stored to computers at a sampling frequency of 1 kHz (MP150, BIOPACK Systems, Santa Barbara, CA, and PowerLab 16/35, ADInstruments‐Japan, Nagoya, Japan) for offline analysis. The data were then sequentially averaged every 1 sec. With two‐dimensional laser speckle flowmetry images, regional facial skin blood flows were obtained every 12 sec from seven different facial regions (right and left infraorbital, subnasal, lips, right and left cheeks, and chin) throughout the experiments (as shown in Fig. 1). The original data of all facial skin blood flows are expressed as arbitrary unit (a.u.) throughout the text and figures and the zero value of the a.u. indicated the absolute zero level of skin blood flow. Regarding exposure to a type of emotionally charged movies, the mean value of each variable obtained over the three consecutive bouts was defined as the response and further averaged among the subjects.

The time course data of the prefrontal NIRS and Doppler skin blood flows were analyzed by using a one‐way ANOVA with repeated measures. If a significant main effect of time was obtained, a Dunnett post hoc test was performed to assess the significant changes from the baseline control. If either normality or equal variance test failed in the one‐way ANOVA, a Friedman repeated measures analysis of variance on ranks with the Dunnett test was performed. With respect to the time course data, the relationships between the variables were assessed by a Pearson's correlation and simple linear regression method (as shown in Fig. 6 and Table 2). Then, for the dependent variable of the changes in right prefrontal Oxy‐Hb, a multiple linear regression analysis was performed (as shown in Table 2).

**Table 2:** Simple and multiple linear regressions of the changes in prefrontal oxygenation, facial and hand skin blood flows, and MAP

(1) Simple linear regression matrix between the variables (summary of correlation coefficients)						
	Right PFC	Left PFC	Forehead	Cheek	Hand	MAP
Right PFC	1.000					
Left PFC	0.995	1.000				
Forehead	0.817	0.799	1.000			
Cheek	0.888	0.867	0.814	1.000		
Hand	0.585	0.573	0.570	0.638	1.000	
MAP	0.562	0.560	0.454	0.509	0.158	1.000

(2) Multiple linear regressions of the right PFC oxygenation from the changes in left PFC oxygenation, facial and hand skin blood flows, and MAP				
(a) Multiple linear regression equation				
Right PFC = −0.0396 + (1.053 × Left PFC) + (0.0570 × Forehead) + (0.144 × Cheek) + (0.00201 × Hand) + (0.00166 × MAP)				
(b) Multiple linear regression table				
	Coefficient	SE	*t*	*P*
Constant	−0.0396	0.00894	−4.425	<0.001
Left PFC	1.053	0.0144	73.046	<0.001
Forehead	0.057	0.0187	3.052	0.003
Cheek	0.144	0.0218	6.628	<0.001
Hand	0.00201	0.0510	0.0394	0.969
MAP	0.00166	0.00404	0.410	0.682

The dependent variable of right PFC can be predicted from a linear combination of the independent variables (left PFC, forehead, and cheek).

PFC, prefrontal cortex oxygenation; Forehead, forehead skin blood flow; Cheek, left cheek skin blood flow; Hand, right hand skin blood flow; MAP, mean arterial blood pressure.

The average responses in the prefrontal NIRS signals, skin blood flows, and systemic cardiovascular variables were compared among the emotional challenges by a one‐way ANOVA with repeated measures and a Student–Newman–Keuls post hoc test. Skin vascular conductance was calculated as a ratio between skin blood flow and MAP and the average responses in skin vascular conductance were also compared among the emotional challenges. The average subjective ratings of pleasantness and consciousness were compared among emotionally charged movies by a Friedman signed‐rank test with the Student–Newman–Keuls post hoc test. The relationship between the two subjective ratings was assessed using the overall data by a Spearman's rank correlation method. Furthermore, the subjective ratings of pleasantness and consciousness determined after every emotional bout were compared with the average responses of the prefrontal NIRS signals and facial and hand skin blood flows during the bout. The relationships between the subjective ratings and the responses were assessed by the Spearman's rank correlation method. A level of statistical significance was defined at *P *<* *0.05 in all cases. All statistical analyses were performed using SigmaPlot^®^ version 13.0 (Systat Software, San Jose, CA). All variables are expressed as means ± SE.

## Results

### Cardiovascular responses during exposures to emotionally charged movies

**Figure 2:**
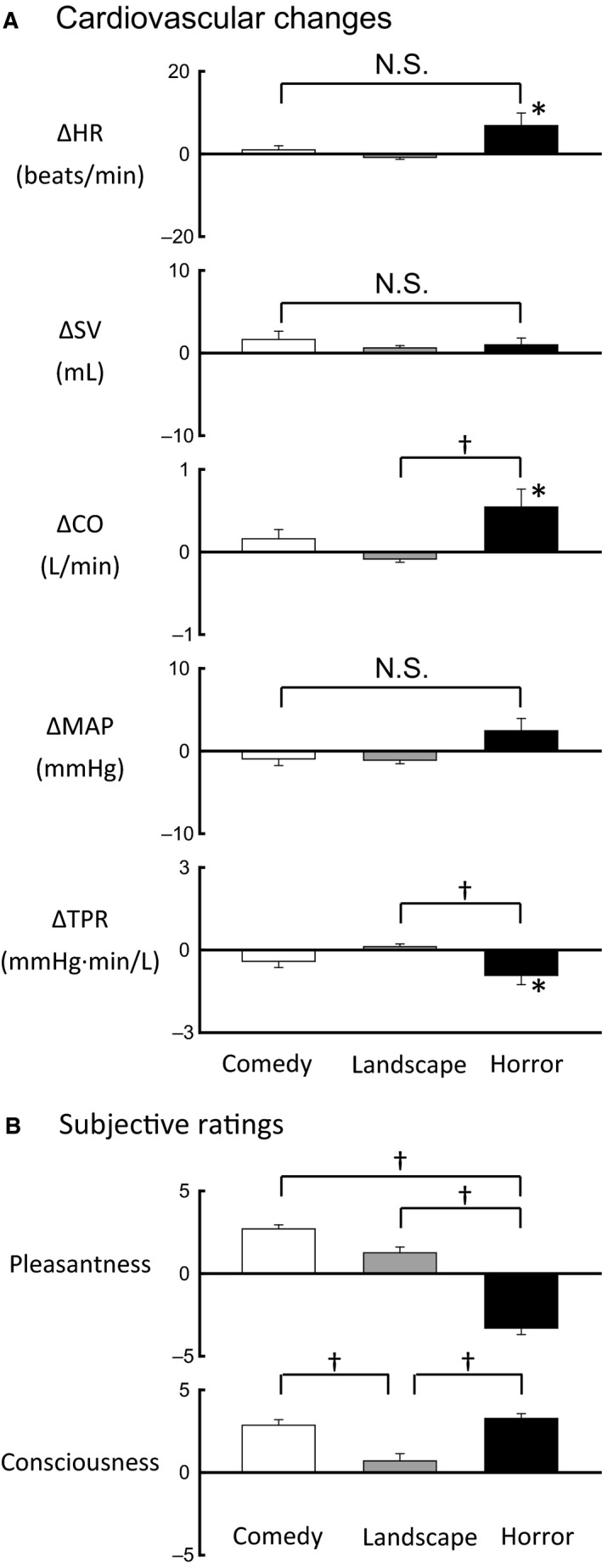
(A) The average responses in heart rate (HR), stroke volume (SV), cardiac output (CO), mean arterial blood pressure (MAP), and total peripheral resistance (TPR) during emotionally charged challenges (comedy, landscape, and horror) in 13 subjects. Each of the emotional interventions consisted of three consecutive bouts involving different movie scenes. The mean data during each intervention, which were collected as the mean value over the three bouts, were further averaged among the subjects. (B) Comparisons of the subjective ratings of pleasantness and consciousness among emotionally charged movies (comedy, landscape, and horror). The subjective ratings collected immediately after every bout of an emotional challenge were averaged among all 14 subjects. *Significant difference (*P* < 0.05) from the baseline control. ^†^Significant difference (*P* < 0.05) between emotional challenges. N.S., not significant between emotional challenges.

The baseline values of systemic cardiovascular variables are shown in Table 1. The cardiovascular responses during exposures to emotionally charged movies are summarized in Figure 2A. The changes in all systemic cardiovascular variables were not statistically significant (*P *>* *0.05) from the baseline control during exposures to the comedy and landscape movie, while HR and CO slightly increased (*P *<* *0.05) and TPR decreased (*P *<* *0.05) during exposure to the horror movie (Fig. 2A). In multiple comparisons among the responses, the increase in CO and the decrease in TRP with the horror movie were greater (*P *<* *0.05) than the changes with the landscape movie.

### Subjective ratings of pleasantness and consciousness

As soon as every bout for an individual movie was completed, the subjective ratings of pleasantness and consciousness were asked against exposure to the emotionally charged movie. As shown in Figure 2B, the pleasantness score was the highest with the comedy movie and the lowest with the horror movie; the difference in the pleasantness score was significant (*P *<* *0.05). On the other hand, the consciousness scores with both comedy and horror movies were higher (*P *<* *0.05) as compared to the landscape movie (Fig. 2B). There was no significant correlation between the extents of pleasantness and consciousness (correlation coefficient [*γ*] = −0.088, *P *=* *0.314 by the Spearman's rank correlation method).

### Responses in regional facial skin blood flow during exposures to emotionally charged movies

**Figure 3:**
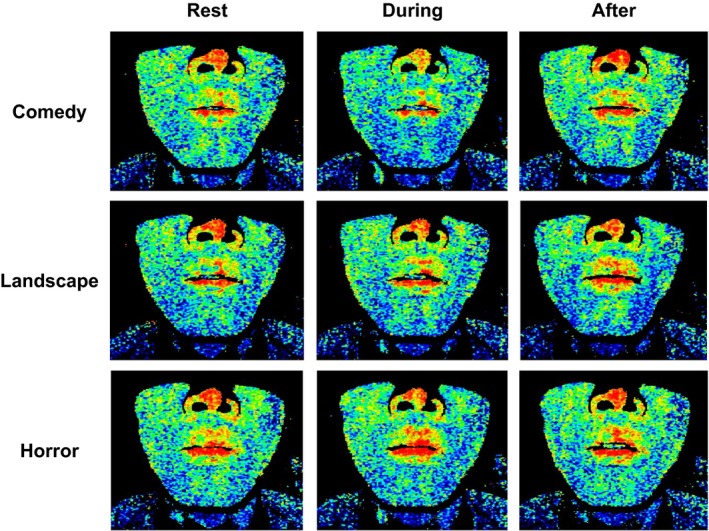
An example of two‐dimensional laser speckle flow measurements of regional facial skin blood flows during exposures to emotionally charged movies (comedy, landscape, and horror) in a subject.

**Figure 4:**
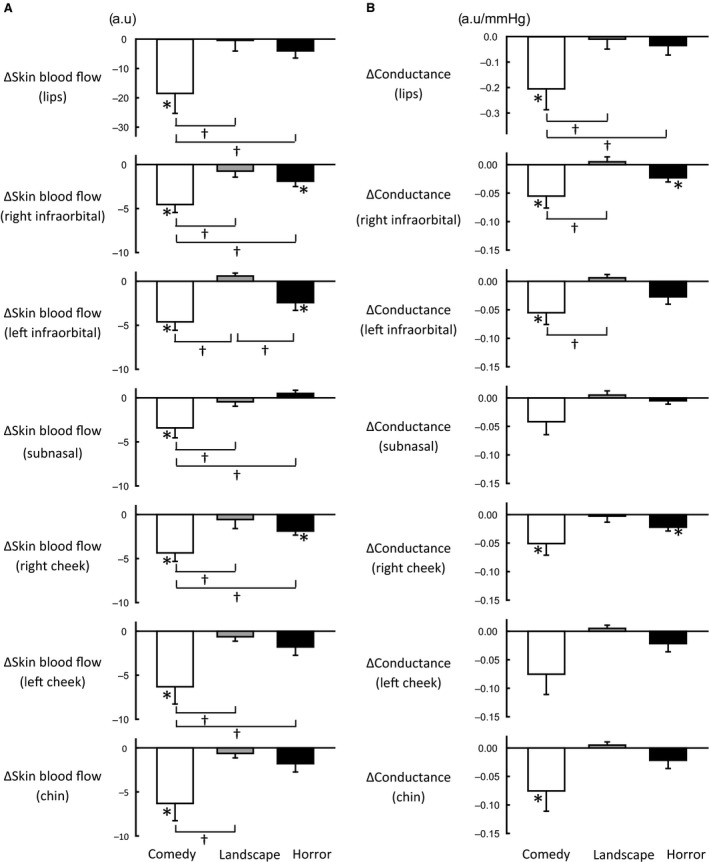
(A) The average changes in regional facial skin blood flows during emotionally charged challenges (comedy, landscape, and horror) in eight subjects. The data during each emotional challenge, which were collected as the mean value over the three consecutive bouts, were further averaged among the subjects. (B) The average changes in regional facial skin vascular conductance during emotionally charged challenges (comedy, landscape, and horror) in eight subjects. Facial skin vascular conductance was calculated as a ratio between facial skin blood flow and MAP. a.u., arbitrary unit. *Significant difference (*P* < 0.05) from the baseline control. ^†^Significant difference (*P* < 0.05) between emotional challenges.

Figure 3 shows an example of the responses in regional facial skin blood flows during exposure to emotionally charged movies in a subject. Evidently, during exposure to the comedy movie, facial skin blood flows decreased, especially in the lips. In contrast, exposure to either horror or landscape movie failed to evoke obvious changes in facial skin blood flow. The average changes in regional facial skin blood flow and vascular conductance during emotional stimulation are compared against the baseline control and also compared among the emotional interventions in Figure 4. All facial skin blood flows decreased (*P *<* *0.05) from the baseline control during exposure to the comedy movie, but failed to change significantly during exposure to the landscape movie. During exposure to the horror movie, some facial skin blood flows (bilateral infraorbital and right cheek) decreased from the baseline. When comparing the average changes in facial skin blood flows, the response magnitudes in the case of the comedy movie were greater (*P *<* *0.05) than those during exposure to the horror and landscape movies (Fig. 4A). The characteristics of the responses in vascular conductance of facial skin regions were almost similar to those of the blood flow responses (Fig. 4B). Several facial skin vascular conductances (lips, bilateral infraorbital, right cheek, and chin) decreased (*P *<* *0.05) from the baseline control during exposure to the comedy movie, while vascular conductance in two facial skin regions (right infraorbital and cheek) decreased (*P *<* *0.05) during exposure to the horror movie. Any facial skin vascular conductance failed to change significantly during viewing to the landscape movie.

### Responses in prefrontal oxygenation during exposures to emotionally charged movies

**Figure 5:**
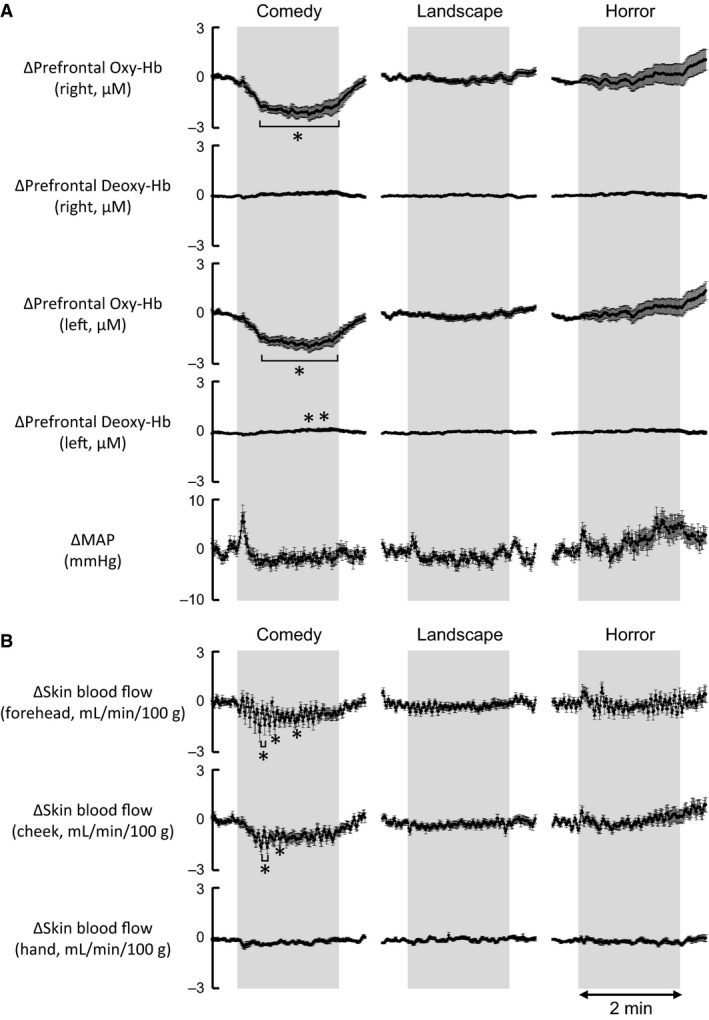
(A) The time courses of the average changes in concentrations of prefrontal oxygenated‐hemoglobin (Oxy‐Hb) and deoxygenated‐hemoglobin (Deoxy‐Hb) on both sides, as well as MAP, during emotionally charged challenges (comedy, landscape, and horror). The mean data during each intervention, which were collected as the mean value over the three bouts, were further averaged among the subjects (*n* = 13–14). (B) The time courses of the average changes in facial skin blood flows (forehead and cheek) and hand skin blood flow, measured with laser Doppler flowmetry, during emotionally charged challenges (comedy, landscape, and horror) in 12–13 subjects. *Significant difference (*P* < 0.05) from the baseline control.

The time courses of the average changes in the prefrontal Oxy‐Hb and Deoxy‐Hb on both sides during exposure to emotionally charged movies are shown in Figure 5A. The bilateral prefrontal Oxy‐Hb decreased (*P *<* *0.05) during exposure to the comedy movie, whereas exposure to either horror or landscape movie failed to cause significant changes in the prefrontal Oxy‐Hb. On the other hand, the Deoxy‐Hb did not change significantly during any exposure to emotionally charged movies, although the left prefrontal Deoxy‐Hb slightly increased during the comedy emotional stimulation. MAP did not change significantly (*P *>* *0.05) during any exposure to emotionally charged movies (Fig. 5A). As shown in Figure 5B, forehead and cheek skin blood flows, measured with laser Doppler flowmetry, decreased (*P *<* *0.05) during exposure to the comedy movie but not horror and landscape movies. Hand skin blood flow did not change significantly (*P *>* *0.05) during any emotional exposure.

**Figure 6:**
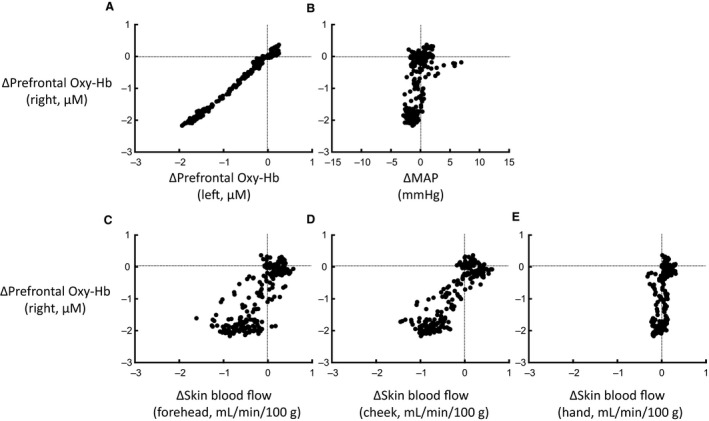
The relationships between the variables in response to the pleasantly charged comedy stimulation were obtained from the data presented in Figure 5. (A) Relationship between the changes in the bilateral prefrontal oxygenation. (B) Relationship between the changes in the right prefrontal oxygenation and MAP. (C) Relationship between the changes in the right prefrontal oxygenation and forehead skin blood flow. (D) Relationship between the changes in the right prefrontal oxygenation and cheek skin blood flow. (E) Relationship between the changes in the right prefrontal oxygenation and hand skin blood flow.

Using the original time course data of the average changes in Figure 5, the relations of the right prefrontal Oxy‐Hb to other variables were analyzed by the Pearson's correlation method (Fig. 6). The correlation coefficient (*γ*) matrix of the simple linear regression between the variables is summarized in Table 2. The changes in the right prefrontal Oxy‐Hb had a highly significant positive correlation with the changes in the left prefrontal Oxy‐Hb (*γ* = 0.995, *P *<* *0.00001). Also, the right prefrontal Oxy‐Hb had a significant positive correlation with the changes in forehead and cheek skin blood flows. The correlation coefficients were 0.817–0.888, greater than the correlation coefficients of 0.562–0.585 in relation to the changes in hand skin blood flow and MAP. For the dependent variable of the changes in right prefrontal Oxy‐Hb, a multiple linear regression was performed (Table 2). As a result, the changes in the right prefrontal Oxy‐Hb could be significantly (*P *<* *0.05) predicted from a significant linear combination (*P *<* *0.05) of the variables of the left prefrontal Oxy‐Hb and forehead and cheek skin blood flows, but not hand skin blood flow and MAP (*P *>* *0.05). It was of interest that the coefficient of 0.144 (*P *<* *0.001) for cheek skin blood flow of the multiple linear regression equation was greater than that of 0.057 (*P *=* *0.003) for forehead skin blood flow.

### Relationships between the subjective ratings and the responses in the prefrontal oxygenation

Whether the responses in the prefrontal oxygenation and facial and hand skin blood flows (measured with laser Doppler flowmetry) were correlated with the subjective rating of pleasantness or consciousness was examined. With respect to pleasantness, significant correlations (*P *<* *0.05) were found with the changes in the prefrontal oxygenation and facial skin blood flows of the forehead and cheek regions (*γ* = 0.24–0.36). However, the changes in hand skin blood flow did not show a significant correlation with the pleasantness score. With respect to consciousness, there were no significant correlations (*P* > 0.05) with the changes in the prefrontal Oxy‐Hb and the facial and hand skin blood flows.

### Responses in prefrontal oxygenation and facial skin blood flow during conversation

**Figure 7:**
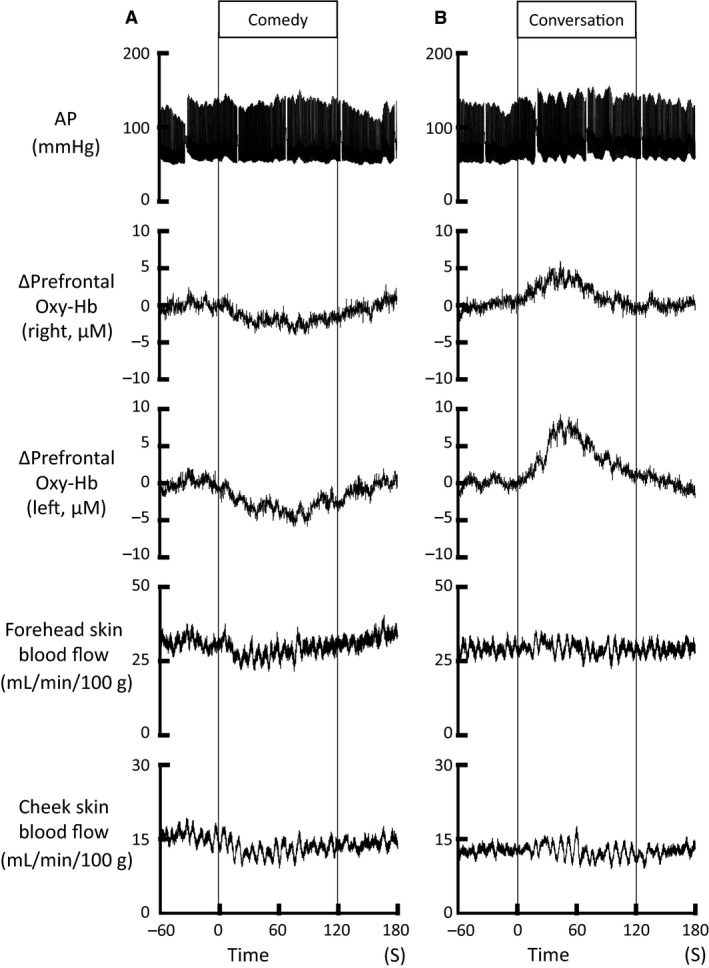
The simultaneous responses in the bilateral prefrontal Oxy‐Hb and forehead and cheek skin blood flow during viewing a comedy movie (A) and during conversation (B) in a subject. Whenever conversation was made by asking the subjective ratings of pleasantness and consciousness, an increase in the prefrontal Oxy‐Hb was observed on both sides, despite no change in the prefrontal Deoxy‐Hb (not shown). On the other hand, both forehead and cheek skin blood flow did not represent substantial changes during conversation, although the facial skin blood flows decreased during viewing a comedy movie, simultaneously with the decrease in the prefrontal Oxy‐Hb.

During viewing a comedy movie, forehead and cheek skin blood flows decreased simultaneously with the decrease in the prefrontal Oxy‐Hb (Fig. 7). On the other hand, whenever conversation was made by asking the subjective ratings of pleasantness and consciousness, an increase in the prefrontal Oxy‐Hb was observed on both sides (as shown in Fig. 7), while the prefrontal Deoxy‐Hb did not change significantly (not shown). Both forehead and cheek skin blood flow, however, failed to represent substantial changes during the conversation.

## Discussion

We have reported that the changes in facial skin blood flow may serve as a more sensitive tool to assess an emotional or mood status in humans (Matsukawa et al. 2017). In this study, we aimed to examine whether prefrontal neural activity has positive correlation with the changes in facial skin blood flow during emotionally charged stimulation. The new findings of this study are (1) that pleasantly charged emotional stimulation (comedy) decreased oxygenation of the bilateral prefrontal cortices, whereas neither negatively charged (horror) or neutral (landscape) emotional stimulation altered prefrontal oxygenation; (2) that the changes in prefrontal oxygenation had a positive correlation with the changes in facial skin blood flows; and (3) that the changes in prefrontal oxygenation and facial skin blood flows were inversely correlated with the subjective rating of pleasantness. Thus, the good temporal correlation between the changes in prefrontal oxygenation and facial skin blood flow led us to speculate that a decrease in prefrontal activity may cause neurally mediated vasoconstriction of facial skin blood vessels.

### Facial skin blood flow as physiological estimate of emotional and/or mood status

To explain quantitatively an emotional or mood status in terms of a physiological variable, the changes in systemic cardiovascular variables and limb skin sympathetic nerve activity have been analyzed in relation to a subjective emotional status so far. Fear‐induced emotional stress or a stress‐induced defensive behavior caused the characteristic pressor response, tachycardia, and cutaneous vasoconstriction (Blair et al. 1959; Adams et al. 1971; Dietz et al. 1994; Vianna and Carrive 2005; Hayashi et al. 2009). Contrary to the data, Brown et al. (2012) reported that exposure to negatively charged pictures did not accompany such cardiovascular response and finger cutaneous vasoconstriction. Matsukawa et al. (2017) found no significant changes in hand skin blood flow and vascular conductance during any of emotional movie challenges (comedy, landscape, and horror), although forearm skin blood flow and vascular conductance decreased during exposure to comedy and/or horror movies. In this study, most systemic cardiovascular variables failed to alter significantly during exposure to any of the emotionally charged movies, although slight increases in HR and CO and a slight decrease in TPR were observed during exposure to a horror movie (Fig. 2). There was the initial small increase in MAP immediately after the onset of comedy stimulation (Fig. 5), which lasted for a brief period of approximately 10 sec and then disappeared throughout the comedy stimulation, although the average response in MAP was not significant from the baseline. Since such brief increase in MAP was also observed at the onset of landscape and horror stimulation, it is likely that the initial pressor response is related to a kind of startle and/or alert reaction evoked by starting audiovisual stimulation. This study confirmed that hand skin blood flow and vascular conductance did not change during any exposure of the comedy, landscape, and horror movies (Fig. 5). Taken together, it is difficult to identify an emotional or mood status in terms of the changes in systemic cardiovascular variables and limb skin blood flow and vascular conductance.

Instead, we have considered regional facial skin blood flow as a physiological candidate for estimating an emotional or mood status (Matsukawa et al. 2017). Regional facial skin blood flow decreased during viewing a positively charged movie, in relation to the subjective rating of pleasantness, whereas negatively charged emotional stimulation failed to elicit a significant response in facial skin blood flows. Since the present study confirmed the above findings about facial skin blood flow using newly recruited subjects, the facial blood flow data are considered quite reproducible and reliable. The decreased facial blood flow was not passively evoked by a decrease in perfusion pressure but was due to active vasoconstriction induced by the autonomic nervous system. Sympathetic postganglionic nerve fibers run along branches of the external and internal carotid arteries and terminate facial skin blood vessels. Activation of facial sympathetic nerves may cause *α*‐adrenergic vasoconstriction of cutaneous blood vessels (Drummond 1994). On the other hand, Nordin (1990) measured skin sympathetic nerve activity in the superior orbital nerve and forehead skin blood flow and found a close temporal relationship between them during a mental arithmetic task, suggesting possible sympathetic vasodilatation of cutaneous blood vessels in the forehead. Facial parasympathetic preganglionic fibers originating from the brain stem contact postganglionic neurons at the otic and pterygopalatine ganglia, whose axons terminate facial skin blood vessels in the lips (Kuchiiwa et al. 1992). Activation of the parasympathetic nerves may cause vasodilatation in facial cutaneous blood vessels (Izumi and Karita 1993). Thus, the decrease in facial skin blood flow during positively charged emotional stimulation may be caused by an increase in sympathetic vasoconstrictor nerve activity and/or decreases in sympathetic and/or parasympathetic vasodilator nerve activity.

### Prefrontal oxygenation during emotionally charged stimulation

A number of studies have reported that the prefrontal cortex has strong relation to emotional recognition and processing. By recording brain activity with PET and fMRI, it has been shown that the ventrolateral and ventromedial subdivisions of the prefrontal cortex are involved in the integration of emotional processing (Wager et al. 2009; Grabenhorst and Rolls 2011; Seo et al. 2014; Kohno et al. 2015; Silvers et al. 2015; Kida and Hoshi 2016; Shiba et al. 2016; Machado and Cantilino 2017). The ventrolateral and ventromedial prefrontal subdivisions were activated during viewing unpleasant pictures or processing negative emotion, whereas the cortical regions were deactivated during viewing pleasant pictures or processing positive emotion. Lesion of the ventromedial prefrontal cortex and stroke of the right middle cerebral artery induced a deficit of emotional recognition (Paradiso et al. 2011; Wolf et al. 2016). On the other hand, noninvasive stimulation with transcranial direct current of the ventromedial prefrontal cortex enhanced processing of pleasant compared to unpleasant scenes (Junghofer et al. 2017). The accumulating evidence suggests that the prefrontal cortex plays an important role in emotional recognition and processing.

NIRS is considered useful as real‐time monitoring of cortical activity by measuring the dynamic changes in regional cerebral oxygenation, even though the spatial resolution is low and the recorded activity is confined to the superficial cortical areas approximately 2 cm below the head surface. The previous NIRS data about the prefrontal activity during emotional interventions are quite controversial (Bendall et al. 2016). The variant observations may result from how to induce emotion (i.e., viewing emotionally charged pictures or facial expressions) and from a substantial difference in the subjective response to a given emotional intervention. Audiovisually elicited emotional stimulation may elicit everyone more consistent pleasant feeling than viewing positively charged images or facial expressions (Matsukawa et al. 2017). The present study showed using this intervention that the prefrontal oxygenation decreased substantially during viewing a comedy movie, whereas it did not change significantly during viewing horror and landscape movies (Figs. 5 and 6). The time course and magnitude of the responses in the prefrontal oxygenation during the emotional interventions were quite similar on both sides. Since the decrease in prefrontal Oxy‐Hb accompanied no significant changes in prefrontal Deoxy‐Hb, the decrease in prefrontal oxygenation is likely to be attributed to a reduction in rCBF. Nevertheless, since both prefrontal oxygenation signal and forehead skin blood flow decreased in parallel during viewing comedy stimulation, there was a possibility that the reduction in prefrontal oxygenation might simply reflect a fall in facial skin blood flow within the illuminated tissue area. To consider the possibility, we examined the simultaneous changes in the prefrontal Oxy‐Hb and forehead skin blood flow during conversation after a bout of emotional stimulation (as shown in Fig. 7), because we know that conversation greatly increases prefrontal oxygenation but does not elicit emotional changes. It was found that the increase in the prefrontal Oxy‐Hb was observed on both sides without accompanying a substantial increase in forehead skin blood flow. Such great dissociation between the responses in prefrontal oxygenation and forehead skin blood flow has been reported during a cognitive Stroop test and voluntary exercise (Endo et al. 2013; Matsukawa et al. 2015; Asahara et al. 2016). The evidence suggests that the prefrontal oxygenation signal recorded in this study predominantly reflects prefrontal rCBF, but not forehead skin blood flow within the illuminated tissue area. The reduction in rCBF was not due to a reduction in perfusion pressure, because MAP did not change during emotional stimulation with a comedy movie (Figs. 2 and 5). Conversely, it is likely that the reduction in rCBF is derived due to redistribution of blood flow within the cerebral cortex, or is associated with vasoconstriction of the regional cerebral blood vessels which may follow deactivation of neurons in the prefrontal cortex.

### Relationship between the changes in prefrontal oxygenation and facial skin blood flow

Previous neurotracing studies have revealed extensive connections from the prefrontal cortex (especially the ventromedial subdivision) to the autonomic nuclei or organs in the rat and monkey (Takagishi and Chiba 1991; Chiba et al. 2001; Gabbott et al. 2005; Levinthal and Strick 2012; Dum et al. 2016). Chemical and electrical stimulation of the prefrontal cortex evokes cardiovascular, respiratory, and metabolic changes (Verberne 1996; Owens and Verberne 2000, 2001; Hassan et al. 2013). Accordingly, if activity of the prefrontal cortex is altered by emotional intervention, a change in prefrontal activity may in turn elicit the autonomic reaction. We found a highly significant correlation between the decreases in prefrontal oxygenation and facial skin blood flow during positively charged emotional stimulation (Fig. 6). On the other hand, the decrease in prefrontal oxygenation had no significant relationship with the changes in hand skin blood flow and MAP. From the relationship of the prefrontal activity to facial or limb skin blood flow, it is considered that autonomic regulation of cutaneous blood vessels is different between the facial and limb regions and that the response in facial skin blood flow may occur in parallel with the response in prefrontal activity during positively charged emotional stimulation, suggesting that the prefrontal cortex may influence vasomotion of facial skin blood vessels through the sympathetic and parasympathetic nervous system. To test the hypothesis, we have conducted a preliminary study to examine the cause–effect of electrical stimulation of the medial prefrontal cortex (mPFC) on skin blood flow of the lower lip in the anesthetized rat. We found that stimulation of the prelimbic area of the mPFC caused a decrease in lip skin blood flow, which seemed abolished following cervical sympathectomy, while stimulation of the infralimbic area of the mPFC caused an increase in lip skin blood flow, which seemed antagonized by atropine (K. Matsukawa and R. Asahara, unpubl. obs.). Thus, activation of the limbic prefrontal cortex may elicit parasympathetically mediated facial skin vasodilatation. These preliminary findings supported at least partly functional connections from the prefrontal cortex to facial skin blood vessels.

### Limitations

Some substantial limitations are involved in this study. First, the two‐dimensional images of skin blood flow in the upper part of the face (including the forehead and eyelids) were lacking due to a technical limitation. Another limitation about the dimensional facial skin blood flow images was low time resolution, because it took 12 sec to scan the lower part of the face with the line‐sensing device. Both limitations were partly compensated by directly measuring forehead and cheek skin blood flows with higher time resolution using laser Doppler flowmetry (Fig. 5). As a matter of fact, we observed that forehead and cheek skin blood flows decreased during viewing a comedy movie and the decreases occurred rapidly. Second, although the movies of the comedy and night landscape provided relatively stationary audiovisual stimulation over the elapsed time period, the horror movie consisted of many variant scenes, which might induce not only fear and negative feeling but also neutral feeling. The audiovisual stimulation by the horror movie seemed not to be always stationary and constant. By this reason, the responses in facial skin blood flow became variable among subjects and even in a given subject. To avoid the nonstationary effect of the horror movie, we should use negatively charged static pictures taken from the database of the international affective picture system in future (Lang et al. 2005). Third, the vascular response in limb skin, by measurement of skin blood flow in the hand dorsum, might be underestimated, because the palmar surface of the hand might be more engaged in emotional vasoconstriction than the dorsal surface of the hand. Although the pattern and time course of the cutaneous vasoconstrictor response to mental arithmetic stress were similar between glabrous and nonglabrous skin (volar hand vs. forearm; volar foot vs. calf), the response magnitude was greater in glabrous skin (Yamazaki et al. 2009; Yano et al. 2009). On the other hand, the increased response in sweating rate in response to the mental stress did not differ between volar foot and calf (Yano et al. 2009) or tended to be greater in glabrous skin (volar hand) than in nonglabrous skin (dorsal hand) (Machado‐Moreira and Taylor 2012). Finally, it has been thought that brain activity is noninvasively monitored as a metabolically evoked change in rCBF, because neural activation in the left primary motor cortex, evoked by transcranial magnetic stimulation, accompanies an increase in rCBF at a latent time of 3–6 sec (Furubayashi et al. 2013). However, there is no way of knowing whether the increase in rCBF involves excitatory or inhibitory neuronal activation or both, even though the change in rCBF may reflect mass neural activity in a local brain region.

## Conclusion

Our laboratory reported that facial skin blood flow may serve as a more sensitive tool to assess an emotional and/or mood status than any of limb skin blood flow and systemic hemodynamic variables. In this study, we found for the first time that both prefrontal oxygenation and facial skin blood flow decreased in parallel during positively charged emotional stimulation, whereas prefrontal oxygenation was augmented during conversation with no substantial changes in facial skin blood flow. Thus, it is speculated that the changes in prefrontal oxygenation does not result from the changes in facial skin blood flow and that the decrease in prefrontal activity may result in neurally mediated vasoconstriction of facial skin blood vessels.

## Conflict of Interest

None declared.
